# Current Analytical Strategies for Antibody–Drug Conjugates in Biomatrices

**DOI:** 10.3390/molecules27196299

**Published:** 2022-09-24

**Authors:** Qiuping Qin, Likun Gong

**Affiliations:** 1 State Key Laboratory of Drug Research, Shanghai Institute of Materia Medica, Chinese Academy of Sciences, Shanghai 201203, China; 2 Department of Immunoassay and Immunochemistry, Center for Drug Safety Evaluation and Research, Shanghai Institute of Materia Medica, Chinese Academy of Sciences, Shanghai 201203, China; 3 University of Chinese Academy of Sciences, Beijing 101408, China; 4 Zhongshan Institute for Drug Discovery, Shanghai Institute of Materia Medica, Chinese Academy of Sciences, Zhongshan 528400, China

**Keywords:** ADC, antibody, payload, LBA, LC–MS, bioanalysis, immunogenicity, interference

## Abstract

Antibody–drug conjugates (ADCs) are a new class of biotherapeutics, consisting of a cytotoxic payload covalently bound to an antibody by a linker. Ligand-binding assay (LBA) and liquid chromatography-mass spectrometry (LC-MS) are the favored techniques for the analysis of ADCs in biomatrices. The goal of our review is to provide current strategies related to a series of bioanalytical assays for pharmacokinetics (PK) and anti-drug antibody (ADA) assessments. Furthermore, the strengths and limitations of LBA and LC-MS platforms are compared. Finally, potential factors that affect the performance of the developed assays are also provided. It is hoped that the review can provide valuable insights to bioanalytical scientists on the use of an integrated analytical strategy involving LBA and LC–MS for the bioanalysis of ADCs and related immunogenicity evaluation.

## 1. Introduction

Antibody-drug conjugates (ADCs) consist of monoclonal antibodies (mAbs), cytotoxic payloads, and linkers that link the mAbs and cytotoxic payloads. ADC is a new type of targeted drugs and most ADCs are directed towards oncology indications where the cancer-targeting abilities of specific antibodies are combined with the cancer-killing abilities of cytotoxic payloads to selectively kill cancer cells [1,2]. It was designed to increase the effectiveness of chemotherapy and reduce its toxicity. Since the first human trial of an ADC therapy performed in 1983 [3], considerable advances have been made in antibody, linker, and payload technologies, leading to the development of ADCs with better efficacy and safety compared to earlier products [4]. By December 2021, a total of 14 ADCs have been approved by the FDA for clinical use and more than 100 ADCs are currently in the different stages of clinical trials [5].

Affinity capture reagents like Protein A/G and target antigen along with immune affinity capture reagents like anti-human IgG and anti-idiotypic antibody have been widely used in the analytical field to extract analytes of interest from biomatrices. In the case of the bioanalysis of ADCs, a ligand-binding assay (LBA) is historically the primary assay platform, but affinity capture or immunocapture have propelled liquid chromatography-mass spectrometry (LC–MS) as a complementary assay technology for PK and ADA assessments [6,7,8,9,10]. As these hybrid assays are the combination of the ligand binding platform and LC–MS technology, we name them as LBA-LC–MS or LBA-LC–MS/MS based assays in this article.

LBA and LC-MS have been the favored bioanalytical techniques for the quantitation of ADCs. For a long period, LBA has been a robust and reliable method choice for quantitation of large molecules in biomatrices. Compared to LBA, LC–MS has been the favored technique for small-molecule quantitation [6]. However, recent advances in the MS instrumentation as well as sample preparation techniques have made LC–MS as a complementary technology for the bioanalysis of protein therapeutics [6,7,8]. As a consequence, it has been reported that multiple LBA, LC-MS, and LBA-LC-MS analytical approaches can be used to quantitatively measure ADC molecules [10,11]. Besides that, bioanalytical methods for pharmacokinetics (PK) and toxicokinetics (TK) should be validated according to related regulatory and industry guidelines [12,13,14].

Like other biological drugs, ADC may elicit anti-drug antibody (ADA) responses. An evaluation of the immune responses to an ADC is important in understanding the PK, safety and efficacy over time. It is thus a regulatory expectation to monitor and characterize the anti-drug antibodies (ADAs) against ADCs [15,16]. Currently, bridging ligand binding assay is the most commonly used format for immunogenicity assessment. The nature of the immunogenicity testing with a bridging assay format is qualitative or semi-quantitative [17,18], and a tiered assay strategy is conventionally adopted for screening, confirming, and tittering ADA levels in biomatrix samples. The same as PK assays, immunogenicity assays should be validated following present regulatory and industry guidelines for biotherapeutics [17,18]. Usually, the samples are first tested with a screening assay, and then the screening-positive samples are tested with a confirmatory assay. Thereafter, the confirmed positive samples are further tested by a tittering assay, a domain-specific assay, and a neutralization assay.

Meanwhile, an LBA-LC–MS/MS approach for immunogenicity testing has been explored in the past few years [9,19,20]. The LBA-LC–MS/MS assay is capable of providing relative concentrations of ADA isotypes in the samples without use of a tired assay strategy and setup of cut-points. Though the assay sensitivity for the LBA-LC–MS/MS assay is currently suboptimal compared with LBA, the LBA-LC–MS/MS assay is a complementary bioanalytical technique for measuring ADA levels and simultaneously providing useful isotype information of ADAs [21]. 

In this review, we aimed to provide current strategies related to a series of bioanalytical assays for PK and ADA assessments. Furthermore, we compared the strengths and limitations of assay platforms with regard to LC-MS and LBA for PK and ADA assays. Finally, we discussed potential factors that affect the performance of the developed assays. We hope that the review can provide valuable insights to bioanalysts on the use of an integrated analytical strategy involving LBA and LC–MS for the bioanalysis of ADCs and related immunogenicity evaluation.

## 2. ADC Forms to Be Quantitated by PK Assays

Due to biotransformation, ADCs can generate different in vivo forms, which may lead to either a change in drug to antibody ratio (DAR) or modifications to the payload/linker [22,23].

If a modification to the payload/linker is negligible, PK assays generally are configured to measure ADCs (all forms of ADCs with DAR > 0), total antibodies (all forms of ADCs including DAR = 0), and released payload [24]. The difference between the ADC and total-antibody profiles theoretically indicates the degree of drug deconjugation. If modifications to the payload/linker are significant, the number of PK assays to be developed will increase. One example given by Myler et al. showed the use of six developed PK assays for a solid tumor-targeting ADC to measure total antibody (T-Ab), active-ADC (antibody conjugated to active payload), total-ADC (antibody conjugated to any payload), Ab conjugated-active payload (conjugated payload), Ab conjugated-payload metabolite (conjugated-payload metabolite), and unconjugated-active payload (unconjugated payload) [22]. Another example given by Faria et al. showed the use of a multiplex approach to quantify total antibody, antibody with unmodified payload, antibody with modified payload, released modified payload, and released unmodified payload [23]. However, the list of potential assays to determine different forms of ADCs could be made on a need-to-know basis or when there are specific mechanistic reasons to do so. Importantly, the ratio of conjugated-payload to total-antibody is the average DAR. DAR is an important parameter reflecting the number of payloads conjugated to the antibody, and its change over time indicates ADC deconjugation and other biotransformation processes including ADC aggregation.

## 3. Assay Platforms

### 3.1. LBA Platform

**Figure 1:**
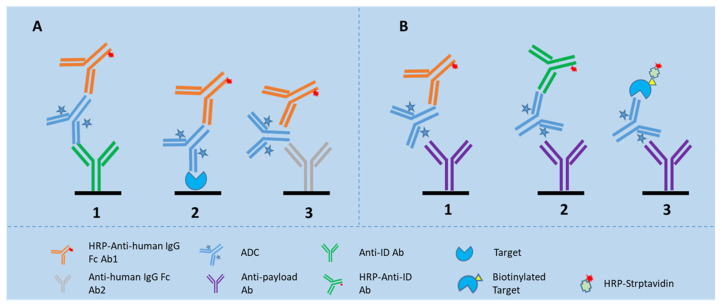
LBA assay formats for detection of TAb and ADC in the biomatrices. (**A**) shows the assay formats for TAb, of which Assay 1 is configured with an anti-idiotypic antibody for capture and an anti-Fc antibody for detection; Assay 2 is configured with the drug target for capture and an anti-Fc antibody for detection; and Assay 3 is a generic assay configured with one anti-Fc antibody for capture and another anti-Fc antibody for detection; (**B**) shows the assay formats for ADC, of which Assay 1 is configured with an anti-toxin antibody for capture and an anti-Fc antibody for detection; Assay 2 configured with an anti-toxin antibody for capture and an anti-idiotypic antibody for detection; and Assay 3 is configured with an anti-toxin antibody for capture and biotinylated drug target together with HRP-labeled streptavidin for detection.

LBA is the favored assay platform for total Ab and ADC measurements because of its adequate sensitivity, good precision, and high sample throughput. A sandwich assay format is usually used, which involves the use of capture and detection reagents that bind specifically to the antibody or payload part of the ADC. For total antibody (TAb), the ADC is commonly captured using an antigen or an anti-idiotypic antibody, and detected by another antibody that binds to a different epitope on the antibody part of the ADC. The latter includes a non-blocking type anti-idiotype antibody or a generic anti-human IgG reagent (see Figure 1A). For total conjugated ADC, the ADC is commonly captured using an antibody against the payload, and detected by an antigen or an anti-idiotypic antibody or a generic antibody against the mAb (See Figure 1B) [25]. Pei et al. used two ELISA methods for the quantification of TAb and ADC of a therapeutic ADC made of an antibody against trophoblast cell surface antigen 2 (TROP-2) and toxins of MMAE in a preclinical study [26]. The ELISA used for TAb was configured with recombinant TROP-2 for capture and HRP-labeled mouse anti-human IgG Fc for detection, whereas the ELISA used for drug-conjugated ADC was configured with a mouse anti-toxin antibody for capture and the same HRP-labeled mouse anti-human IgG Fc for detection. In case the payload of an ADC undergoes in vivo biotransformation to an inactive metabolite, the PK profile of active ADC can also be determined by LBA with a specific reagent that solely binds to the active payload. For example, Myler et al. developed and validated a method using Meso Scale Discovery (MSD) platform for the quantitation of active ADC [22]. However, in the second example, an LC-MS approach seems more practical than an LBA approach, as the specific reagent useful for detection of the biotransformed payload is usually hard to generate.

For ADCs made with humanized monoclonal antibodies (mAbs) in animal studies, a generic LBA method configured with general anti-human immunoglobulin antibodies as the capture and detection reagents for total antibody can be used for the pharmacokinetic evaluation (see Figure 1A3). A generic ELISA assay strategy is useful particularly at an early stage of drug development when custom ELISA reagents, such as recombinant antigens or anti-idiotypic antibodies are most likely unavailable [25]. Later on, quantifying total antibody requires the use of a selective reagent specific for the antibody of interest.

### 3.2. LC-MS Platform

#### 3.2.1. LC-MS/MS

**Figure 2:**
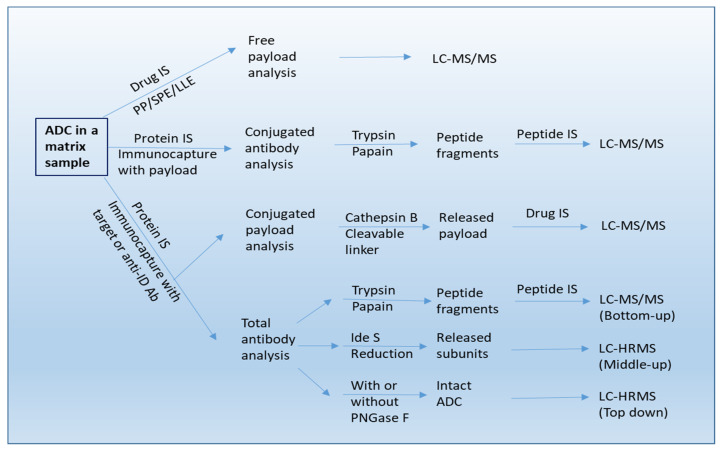
A workflow of bioanalysis for ADCs in biological sample using LC-MS/MS, LBA-LC/MS/MS, and LBA-LC/HRMS methods. IS: internal standard. Anti-ID Ab: anti-idiotypic antibody.

LC-MS/MS is generally viewed as the most appropriate platform for small-molecule analysis. For ADC, it is conventionally used for small-molecule unconjugated payload analysis. Due to the high hydrophobicity of cytotoxins, the quantitation of deconjugated payload in circulation is typically achieved by protein precipitation (PP) or solid-phase extraction (SPE) or liquid–liquid extraction (LLE) of analyte from biomatrices followed by LC–MS/MS analysis with a multiple reaction monitoring (MRM) mode (see Figure 2) [25]. Furthermore, the LC–MS/MS platform also allows for analysis of the small-molecule metabolites.

However, owing to the distinct release mechanisms, deconjugation of an ADC with a non-cleavable linker produces different forms of unconjugated payload when compared to that of an ADC with a cleavable linker. For ADCs with non-cleavable linkers, several payload-containing forms including free payload may be produced, which can be quantified as well [27]. For example, Kaur et al. quantified DM1, Lys-mcc-DM1, and mcc-DM1 in the PK and TK studies of T-DM1 [25].

#### 3.2.2. LBA-LC-MS/MS

For ADCs with cleavable linkers, the conjugated-payload is predominately measured by affinity capture or immunocapture LC–MS/MS assays [28]. Conjugated payload is quantified after isolation of small molecules from linker cleavage of captured ADCs. For example, for the ADC utilizing an enzyme-sensitive dipeptide Val-Cit linker, the release of payload can be achieved by digestion with proteases such as cathepsin B and papain [10,28,29,30]. For the ADC with a disulfide bond linker, a reducing agent of dithiothreitol (DTT) or Tris(2-carboxyethyl)phosphine (TCEP) is employed for cleavage [28]. Immuno-affinity capture involves the use of a biotinylated specific anti-idiotypic antibody or target antigen, which is immobilized on streptavidin-coated beads or cartridges. The ADC in the biomatrix is then captured and followed by an elution step. Thereafter, the eluted ADC is specifically cleaved at the linker site by a lysosomal enzyme, and finally the payload released from the ADC can be analyzed by LC–MS/MS (see Figure 2). 

Of note, protein A or protein G or anti-human IgG can be used for the analysis of conjugated-payload particularly in the early development and non-clinical studies [10,24,31,32]. In addition, payloads conjugated by non-cleavable linkers are often indirectly determined by multiplying in vivo average DAR value with total antibody concentration [29]. The measurement of conjugated-payload provides information on the number of the cytotoxic payload linked to the antibody.

LBA-LC-MS/MS can also be used for TAb and ADC quantitation, especially during early discovery when appropriate LBA reagents are unavailable [33,34]. A bottom-up LC-MS strategy is used for quantitative determination of total antibody and ADC (see Figure 2). For TAb quantitation, the ADCs are first extracted from the biomatrices by affinity capture with an anti-idiotypic antibody or generic capture reagents like anti-human IgG against the mAb component of the ADC. The affinity enriched ADCs are denatured/reduced, alkylated, and then digested with an enzyme, and followed by addition of a stable isotope-labeled internal standard and quantification of a signature peptide selected from complementarity-determining region (CDR) by LC–MS/MS [32,33,34,35]. It is worth noting that a stable-isotope-labeled antibody as internal standard can also be incorporated from the beginning of the enrichment process [36,37]. For ADC quantification, the sample enriched by immunocapture with an anti-payload antibody is digested and then the antibody is quantified by using the same strategy as that is used for quantification of the total Ab [31,32,33,34,35].

A generic LBA-LC-MS/MS method for TAb can be configured with general anti-human IgG Fc antibodies for capture of ADCs from animal plasma, and signature peptides from the constant region of antibody part for detection. Several Fc region peptides unique to the human immunoglobulin framework have been selected as detection signature peptides for animal studies [37,38] 

#### 3.2.3. LBA-LC-HRMS (High-Resolution Mass Spectrometry)

A combination of affinity capture with HRMS based intact mass analysis provides an accurate method for characterization of DAR at an intact protein/subunit level [39]. This involves deconvoluting the mass spectra to a series of “zero-charge” masses, and then computing average DAR by integrating and weighting the spectral peak area or peak intensities. In addition, the DAR distribution at different time points can also be determined with this approach. For example, Kaur et al. showed that all DARs were captured by using biotinylated HER2 immobilized onto streptavidin-coated paramagnetic beads [25]. After washing, the bead-bound ADC forms with all DARs were first deglycosylated with PNGase F and then eluted with 30% acetonitrile in water containing 1% formic acid for LC–MS analysis involving the use of a polymeric reversed-phase column (Agilent PLRP-S column). Analytes were ionized by electron spray ionization (ESI) and detected by a Q-Star^®^ XL mass spectrometer (AB Sciex) operated in the positive TOF (Time of Flight)-MS mode. Raw data were deconvoluted using Analyst QS 1.1 software (Applied Biosystems, Waltham, MA, USA), and peak areas were obtained for each DAR of interest. Relative intensities for the DARs were calculated. In this way, the affinity capture LC–HRMS method provided direct measurement of DARs in vivo. Additionally, this “top-down” strategy also enabled the quantification of intact ADC from the biomatrix (see Figure 2) [40] and the characterization of in vivo biotransformation of ADCs in circulation [41]. 

## 4. LBA vs. LC-MS Based PK Assays

For in vivo analysis, quantification of ADCs in plasma and serum involves LBA or LC-MS based methods, or a combination of the two methods. In general, LBA based methods are more commonly used in quantification of ADCs in plasma and serum than LC-MS based methods. LBA methods offer high throughput and low equipment cost and have been used in the pharmacokinetic assessments for several approved ADCs. However, LBA based methods are often species-dependent, and related method development in most cases needs specialized reagents and is time-consuming, which is the situation encountered in the early phase of ADC development and the reason why a generic method is of value to use. Another feature for LBA based methods is that they cannot differentiate ADCs with different DAR values. By comparison, LC-MS based assays are matrix- and species independent. When the structural and biotransformation information is not ready, or critical reagents are unavailable, LC-MS methods are preferred as their development does not use specialized reagents and consumes less time. Furthermore, owing to their high specificity of molecular-resolution, LC-MS-based methods are able to determine DARs in biological samples directly, facilitating the characterization of ADC forms with different DAR values. However, it should be noted that no regulatory guidance is currently available for LBA-LC–MS based assays for ADCs [42].

## 5. Detection of ADAs

In general, ADAs elicited for an ADC includes those specific to intact ADC, to linker, to payload, and to the unconjugated antibody. All the ADAs generated against the ADC should be properly detected [43]. Usually, the entire ADC molecule is used in an ADA assay, enabling identification of ADAs with specificity for any component of the intact ADC. 

### 5.1. Assay Platforms

#### 5.1.1. LBA Platform

**Figure 3:**
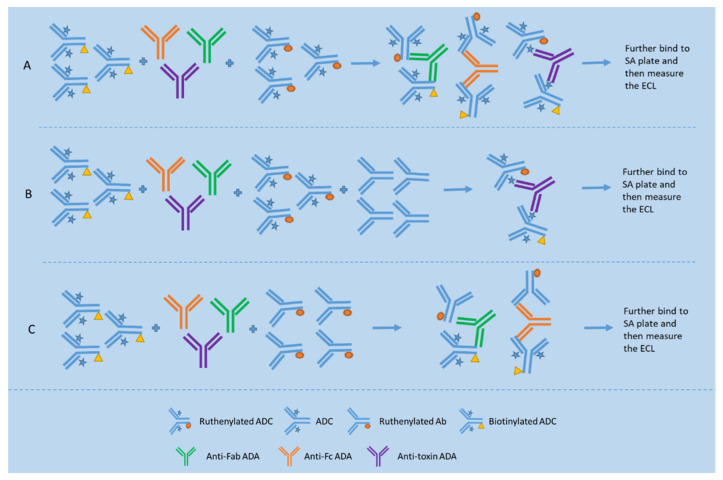
Bridging assay format for total ADA detection and two assay formats, namely epitope competition method and epitope detection method for domain specificity testing. (**A**) shows a typical bridging format for detection of ADAs against epitopes located on different portions of the ADC; (**B**) shows the epitope competition method used for detection of ADAs reactive with the antibody portion of the ADC based on the inhibition signal obtained when compared to the signal of the assay depicted in Figure (**A**); (**C**) shows the epitope detection method used for detection of ADAs reactive with the antibody portion of the ADC based on the specific binding signal obtained.

Presently, bridging ligand binding assay (Bridging LBA) is the format most commonly used in immunogenicity testing [15,16,43,44] where a biotinylated drug is utilized for capturing ADAs, and ruthenium or HRP-labeled drug and even digoxigenin (DIG)-labeled drug for detecting the bridged ADA–drug complex (see Figure 3A). Other assay formats used in immunogenicity testing include direct and indirect assay formats. The direct assay format involves the use of drug or biotinylated drug for capturing ADAs, and anti-IgG species-specific antibodies for detecting ADAs. The indirect assay format involves the use of drug-specific mAb for capturing ADA-bound and unbound drug, and anti-IgG species-specific antibodies for detection of ADAs (the drug must be free of testing species Ig Fc). 

A tiered assay strategy is conventionally used to screen, confirm, and titrate ADA levels in the samples. A screening-assay cut point is established statistically based on the response signals from a relatively large population (*n* ≥ 30 for nonclinical studies and *n* ≥ 50 for clinical studies) of drug-naive subjects and is used to determine screening-positive samples (95% confidence level). Those screening positive samples are then subjected to a confirmatory assay where a competition format is used to confirm drug specificity (99% confidence level). Thereafter, the confirmed positive samples are further analyzed by a titration assay. Based on a thorough risk assessment, confirmed positive samples can further be characterized for domain specificity and, in later phases of development, a neutralizing antibody (NAb) assay may be used to further assess the impact of ADAs [45].

Two approaches are used for domain specificity testing, namely the epitope competition approach and epitope detection approach (see Figure 3). The epitope competition approach is capable of identifying reactivity with the linker/small-molecule drug in an ADC (see Figure 3B), while the epitope detection approach is sensitive to minor components of a polyclonal antibody response (see Figure 3C) [45]. For example, Hoofring et al. demonstrated that, with surrogate controls, detection of a minor anti-linker/toxin or anti-mAb ADA was only possible with the epitope detection approach [17].

Two assay formats have been used to measure NAb activity, cell-based bioassays, and non-cell-based competitive LBA. Selection of the appropriate assay format should be reflective of the therapeutic mechanism of action (MoA). Due to the MoA of ADCs, cell-based NAb assays are the regulatory authorities’ preferred method to detect the presence of NAbs [16]. These assays use cellular responses as an assay end point to detect NAb-mediated inhibition of the biological function of the ADC and are considered more reflective of the in vivo situation than LBA. The use of LBA is considered appropriate only if efforts to develop a reliable cell-based assay are not successful due to susceptibility of the cells to matrix or drug interference or the related MoA does not involve cells at all [16]. 

To simplify ADA assay development during nonclinical studies, generic ADA ELISAs have been used to measure immunogenicity of humanized antibody therapeutics in mouse and cynomolgus monkey studies [46,47]. These assays used anti-human constant region antibodies to capture the drug and anti-mouse or anti-cynomolgus IgG species-specific antibodies to detect the drug-ADA complex. 

Generic ADA electrochemiluminescence (ECL) immunoassays have also been developed for nonclinical studies in three animal species: mouse, rat, and cynomolgus monkey. The format of these assays was to coat the therapeutic on carbon surface plates to capture ADA, which was then detected with a species-specific antibody [48].

#### 5.1.2. LBA-LC-MS/MS Platform

##### Direct LBA-LC-MS/MS Assay Format

**Figure 4:**
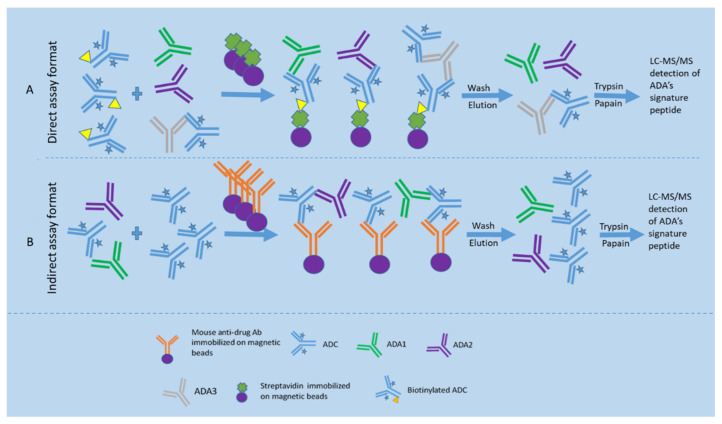
Direct and indirect LBA-LC-MS/MS assay formats for ADA detection. (**A**) shows the direct LBA-LC-MS/MS assay format for detection of ADAs, where samples are pre-incubated with the biotinylated drug to form a biotinylated drug-ADA complex and then captured by the streptavidin magnetic beads. Subsequently, nondrug-specific proteins are removed from the beads by washes. The bound ADAs are eluted by an acidic buffer and followed by neutralization. Thereafter, the eluted antibodies undergo reduction, alkylation, and digestion with trypsin to obtain ADA tryptic peptides. The signature peptides derived from ADAs are chromatographically separated and then detected by measuring each signature peptide under a specific SRM mode; (**B**) shows the indirect LBA-LC-MS/MS assay format for detection of ADAs, where excess drugs are spiked into the serum sample to saturate the ADA binding sites, followed by using a mouse anti-drug monoclonal antibody immobilized on magnetic beads to capture the ADA-drug complex. Thereafter, the ADAs are directly measured by the ADA’s signature peptides.

In the direct LBA-LC-MS/MS assay format (see Figure 4), ADA samples are firstly pre-incubated with the biotinylated drug to form a biotinylated drug-ADA complex and then captured by the streptavidin magnetic beads. Subsequently, nondrug-specific proteins are removed from the beads by buffer washes. The bound ADAs are eluted by an acidic buffer at pH 2–3 and followed by neutralization to pH 8. Thereafter, the eluted antibodies undergo reduction with DTT, alkylation with iodoacetamide, and digestion with trypsin to obtain ADA tryptic peptides. The signature peptides derived from ADAs are chromatographically separated and then detected by measuring each signature peptide under a specific selected reaction monitoring (SRM) mode. Importantly, calibration curve samples are prepared with the purified Ig isotypes or subclasses (IgG1, IgG2, IgG3, IgG4, IgM, IgE, IgD, IgA1, and/or IgA2) in a mixture of elution/neutralization buffer, and then undergo the same sample processing steps as unknown samples except for the affinity capture process. The LC–MS response of each signature peptide for a specific SRM is proportional to the concentration of the corresponding intact Ig isoform, and the chromatographic peak area ratio of the signature peptide to the internal standard is used for the quantitation. The concentrations of each Ig isotype in an unknown sample are back-calculated from its signature peptide response using the established calibration curve [49]. It is worth noting that the elution step is performed with an acidic buffer (pH 3) as it does not disrupt the biotin/streptavidin interaction and only has a minimal contribution to the detection [45].

##### Indirect LBA-LC-MS/MS Assay Format

For the indirect LBA-LC-MS/MS assay format (see Figure 4), excess drugs are spiked into the serum sample to saturate the ADA binding sites, followed by using a mouse monoclonal antibody against the drug to capture the ADA-drug complex. Thereafter, the bound ADAs are measured using the universal peptides specific to each isotype/subclass [45,50]. 

The LBA-LC–MS/MS for immunogenicity testing is currently regarded as a semi-quantitative assay with acceptable specificity, sufficient drug tolerance, and capability of multiplexed detection for ADA isotyping [51]. As the LBA-LC–MS/MS assay provides relative concentrations of ADA isotypes in the samples, the tiered approach is unneeded.

## 6. LBA vs. LBA-LC-MS/MS Based ADA Assays

LBA is more commonly used in detection of ADAs in plasma and serum but is often matrix-dependent. The assay sensitivity of LBAs is superior to that of LBA-LC-MS/MS based assays no matter what format is used. The LBA-LC-MS/MS based assays are complementary to the LBA, able to provide a relative ADA level and isotype information [9] LBA-LC–MS/MS assays can overcome some unique challenges that cannot easily be overcome by LBA, and serve as a confirmatory test for ADA positive samples. Direct LC–MS/MS detection of unique signature peptides from ADA molecules allows increased assay specificity to be obtained, which may resolve limitations of some LBAs. In the direct assay format, the use of a high concentration of biotinylated drug for immunocapture combined with independence from bivalent binding for capture and detection reduces drug and drug target interference. However, the immunocapture efficiency of the indirect assay format is still subject to interference with the drug in the samples [49]. It is worth noting that the current immunogenicity testing guidelines have been based on the LBA, whereas no guideline is available for LBA-LC–MS/MS platform-based ADA assays. In addition, some other features seen with the PK assays are likely present with the ADA assays (see the LBA vs. LC-MS based PK assays section).

## 7. Factors That Interfere with the Assay Performance

### 7.1. PK Assays

#### 7.1.1. DAR

After drug administration, DAR in vivo changes with time due to the deconjugation or biotransformation. As a consequence, the ADC reference standard for quantitative assays may not be identical to the ADC mixture in the in vivo study samples, especially at later PK time points. If the antibodies used in the assays for TAb and ADC are affected by DARs, then the concentrations of TAb and ADC may not represent the real concentrations of the analytes, thereby affecting interpretation of the study data. 

Kaur et al. reported a case study on characterization of a LBA for an anti-STEAP1 ADC. The assay used an anti-drug mAb for capture and a biotinylated anti-CDR mAb and streptavidin-HRP for detection. It was shown that the ADC ELISA did not measure DAR1 [25]. Thus, ADC concentration in the sample may be significantly underestimated if DAR1 is present in a high relative abundance. There is another report given by Stephan et al., showing that ADC conjugates with various DAR values performed differently in the total and conjugated antibody assays [52]. Therefore, it is important to evaluate the performance of TAb and ADC assays with all DARs expected in vivo regardless of assay platforms. To prevent this phenomenon from occurring, DAR-insensitive assays that allow for measuring various DAR components of the ADC equally should be built, thereby resulting in assays not biased toward varying DAR values of the ADC.

**Figure 5:**
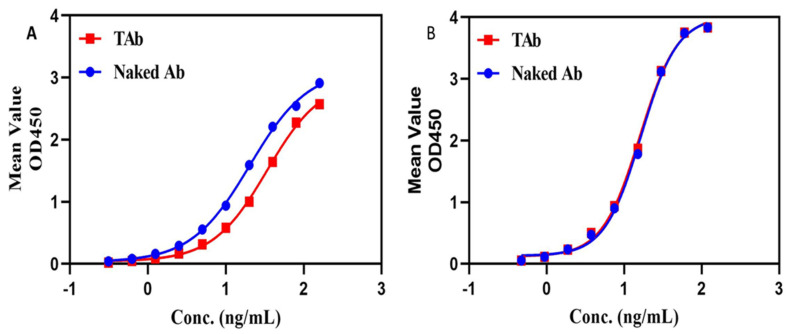
DAR effect on TAb assays. One TAb assay was configured with target antigen for capture and an HRP-labeled polyclonal antibody for detection, but the assay gave higher binding signals for the drug unconjugated antibody than for the TAb (see (**A**)). Another TAb assay was configured with the same target antigen for capture but with a HRP-labeled monoclonal anti-Fc antibody for detection, and the assay gave almost the same binding signals for the drug unconjugated antibody and for the TAb (see (**B**)). The blue line represents the drug unconjugated antibody, namely naked antibody, and the red line represents the TAb.

We once configured a total antibody assay for a HER2-targeting ADC which was site-specifically conjugated with eribulin (DAR = 4). The assay was initially configured with HER2 as the capture reagent and HRP-labeled goat anti-human IgG as the detection reagent. The assay was then used to analyze the unconjugated antibody and TAb. As can be seen in Figure 5A, the specific binding signal levels at various concentrations were all higher for the unconjugated antibody than for the TAb, indicating that the toxin molecules on the ADC could cause steric hindrance to the binding of the labeled antibody. We thereafter replaced the HRP-labeled detection reagent with a monoclonal anti-Fc antibody, and the resulting assay performed equally well for both the TAb and the unconjugated antibody, indicating that the small toxins conjugated to the antibody do not affect the binding of the labeled monoclonal antibody to the ADC (see Figure 5B). Thus, the latter assay was picked up for the validation and subsequent sample bioanalysis.

#### 7.1.2. ADA

ADA response is in principle a polyclonal response, directed to different epitopes and featured with diverse ADA concentrations and affinities as well as varying antibody isotypes and subtypes [53]. ADA can be classified into two categories, namely neutralizing and binding ADA. The neutralizing ADA binds to the active site of the antibody portion of the ADC, while binding ADA binds to other portions of the ADC. Both binding and neutralizing ADA have the potential to impact the PK assays. The neutralizing ADA can compromise the capture or binding efficiency of the target antigen or anti-idiotypic antibody, leading to underestimated concentrations of ADCs. The binding ADA may compete with the labeled detection reagent to bind to the same epitope on the ADC, resulting in reduced response signal. Furthermore, the binding ADA may also affect the capture or binding efficiency of the target antigen or anti-idiotypic antibody due to the related epitope which is linearly or conformationally in close proximity to the target binding site. 

#### 7.1.3. Soluble Target or Extracellular Domain of the Target

When a target antigen or an anti-idiotypic antibody is used for enrichment or binding of an ADC in the PK assays, soluble target interference should be evaluated. It is the intact form of the ADC that is detected by the PK assay. The soluble target, if available in a sufficient amount, can bind to the ADC, which may compromise the capture or binding efficiency of the immobilized antigen or anti-idiotypic antibody [10], leading to underestimated concentrations of ADCs.

#### 7.1.4. Sample Preparation

In LC-MS/MS based methods, sample preparation and charge states can affect the quantification of the analytes. Due to the high hydrophobicity of typical cytotoxins, LLE and SPE are often used for extraction. Importantly, sample preparation-related deconjugation of the payload should be avoided. For example, to avoid deconjugation, the pH should be adjusted for acid-labile linkers such as the acid-cleavable hydrazone linker and benzyl carbonate bond-based linker; protease inhibitors may be added to avoid linker cleavage caused by proteases present in the sample, and sample extraction is better performed in an ice-water bath to minimize deconjugation [36,54,55,56,57]. In addition, the reactive thiol groups of maytansinoid payloads may undergo disulfide exchange with other thiol-containing molecules in the matrix, leading to the formation of a dimer after release [41,54]. Therefore, reduction and derivatization of thiol are usually performed before LC-MS analysis of this type of payload [54]. 

### 7.2. ADA Assays

#### 7.2.1. Drug Target

It is necessary to identify and mitigate the interference mediated by the presence of soluble drug target. A drug target, when present at sufficiently high concentrations in the bloodstream, may interfere with the performance of ADA and NAb assays, leading to either false-positive or, in some cases, false-negative ADA and NAb assay results [58]. Related mitigation approaches include the use of anti-target antibodies [59], soluble versions of the receptors [60], target-binding proteins [61], lectins [62], and solid-phase removal of targets. For example, Wang et al. used polyclonal anti-soluble B-cell maturation antigen (sBCMA) antibodies as scavengers to deplete sBCMA at a concentration four times greater than the median level of this tumor-associated antigen observed in the multiple myeloma patient population, thereby mitigating sBCMA interference in the related functional assay for the detection of neutralizing antibodies against a BCMA-CD3 bispecific antibody [59]. For another example, Dengler et al. managed to overcome the interference of prior checkpoint inhibitor with the immunogenicity assays by using antibodies specific to pembrolizumab or nivolumab [63].

#### 7.2.2. Drug

Bridging immunoassays for detection of ADAs is susceptible to interference from circulating drug [64]. In the bridging assays, the drug is labeled separately with different labeled molecules such as biotin and sulfo-tag, and some anti-drug antibodies present in the sample will form a bridge between the two labeled molecules. The drug, when present at sufficiently high concentrations in the bloodstream, can compete with the labeled drug to bind to ADAs and reduce the signal of the ADA assay, consequently leading to false negative results. It is worth noting that cell-based NAb assays are vulnerable to drug interference as well.

**Figure 6:**
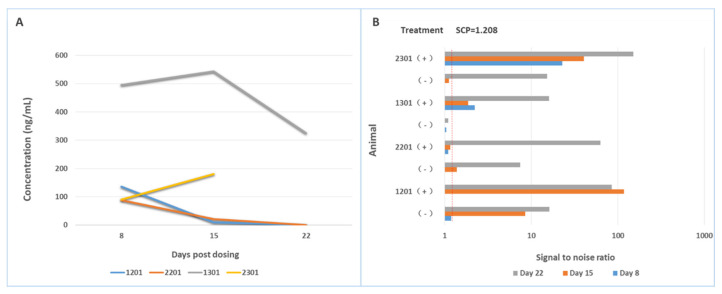
Drug interference on the bridging assay is mitigated by removal of free drug in the sample. (**A**) shows the concentrations of drugs in animal (cynomolgous monkey) samples collected at different times; (**B**) shows the signal-to-noise ratios of each sample determined with a screening assay for ADA either in the presence of a sample pre-treatment step or in the absence of a sample pre-treatment step. After removal of free drug from the samples, ADA positive animals are detected earlier and with higher ADA titers.

Various mitigation approaches have been used to overcome drug interference with the ADA assays, which include the use of acid dissociation [65], affinity capture elution (ACE) [66], solid-phase extraction with acid dissociation (SPEAD) [67], bead-extraction and heat-dissociation (BEHD) [68], precipitation and acid dissociation (PandA) [69], bead extraction with acid dissociation (BEAD) [70], and so on. Acid dissociation and solid-phase extraction are commonly incorporated in the ADA assays to reduce drug interference occurred in the study samples and prevent underestimation of ADA response. For example, Niu et al. managed to overcome matrix and drug interference by combining a biotin-drug extraction with acid dissociation (BEAD) procedure into an ECL direct ADA assay [70]. The developed ADA assay utilized two-step acid dissociation and excess biotin-drug on magnetic streptavidin beads to extract total ADA, which was further captured by soluble biotin-drug and detected in an ECL semi-homogeneous direct assay format. The BEAD procedure eliminated interference by serum matrix and free drug, and enhanced assay sensitivity. We used Sepharose beads conjugated with a monkey-absorbed polyclonal anti-human IgG to remove free drugs from monkey serum samples collected from a single dose PK study, and found ADA could be detected early on Day 8 post dosing, and, furthermore, a greater number of ADA-positive samples with higher titer results were obtained (see Figure 6) [71]. 

#### 7.2.3. Modification of Critical Reagents

ADA methods require conjugation of the ADC to labels for capture and/or detection, which may be problematic for an ADC as the labeling modification may alter its immunologic reactivity. The antibody part of an ADC has undergone one round (or more) of conjugation to a small-molecule drug. If both conjugation and labeling chemistry utilize amine groups of lysine residues, the net positive charge of the doubly labeled ADC reagent may be reduced. As a result, the solubility of these reagents is likely to differ from the parent mAb when the additional label is a hydrophobic biotin molecule or sulfo-tag molecule. Furthermore, the ADC’s stability may change in response to the labeling modification. Lastly, sites for conjugation within the CDRs may be modified, and the ability of critical reagents to detect ADA that target the modification regions could be compromised by high levels of labeling. Therefore, careful assay design and selection of assay platform and critical reagents are required for optimal detection of ADAs against all components of the ADC [72,73].

#### 7.2.4. Preexisting Antibodies

An alternative to the qualitative screening assay approach can be used to assess the quantity and quality of ADA when pre-existing antibodies are present. In the presence of pre-existing antibodies, if the titer of the antibodies increases after exposure to the ADC, then they can be reported as treatment-boosted to differentiate them from treatment-induced antibody titers. For example, a boosted ADA response may be defined as a titer that is two dilution steps greater than the pre-treatment titer, when twofold dilutions are used to determine the titer [16].

#### 7.2.5. Rheumatoid Factor (RF)

RF is generally an IgM antibody that can bind Fc regions of an IgG, thereby forming a bridge between the two labeled ADC molecules and generating a false positive response. However, RF interference can be mitigated by adding aspartame or Asp-Phe or its amidated derivative and CaCl_2_ to the binding buffer [74]. Added aspartame binds to the Fc of a human IgG antibody, which will inhibit the RF reactivity with the Fc. In addition, in clinical settings, an assay specific for the non-Fc region of the ADCs rather than against the intact ADCs can be developed to circumvent RF interference.

## 8. Conclusions

ADCs are generally complex and structurally heterogeneous mixtures, typically containing numerous product-related forms resulting from the continuous deconjugation and biotransformation in vivo [75,76,77]. Meanwhile, various analytical platforms are available [78], of which an integrated platform consisting of LBA, LC-MS, LBA-LC-MS, and HRMS is particularly useful for better understanding of the pharmacokinetics, metabolism, and biodistribution of ADCs. To date, a number of bioanalytical strategies have been employed to build PK and ADA assays in the early stage of drug development, nonclinical and clinical studies. However, current protein-level enrichment only works well in plasma and serum but not tissues, which limits the bioanalytical methods only applicable to plasma and serum matrices [79]. Moreover, selection of the product-related forms for analysis should reflect the purpose of the study, and subsequently the bioanalytical strategies tailored to their molecular properties as well as analytical methods accordingly developed should be capable of measuring the selected analytes. Additionally, a bioanalytical strategy employed for each ADC form should be generated mainly on the following bases, namely available assay platforms, critical reagents, biomatrices, nature of the study, and drug development phase of assay application. It is worth noting that no regulatory guidance is currently available for LBA-LC–MS/MS based PK and ADA assays; nevertheless, the application of any assay developed on this platform in the regulated studies requires the assay to be validated prior to use [28,80]. Finally, with evolving technologies, future ADCs may engage the use of bispecific antibodies, dual-payloads, and immune-stimulating payloads [81]. It is therefore expected that novel analytical strategies will be developed to address emerging challenges brought about by the new modalities.

## Data Availability

No new data were created in this study. Data sharing is not applicable to this article.
